# Pain REduction with bone metastases STereotactic radiotherapy (PREST): A phase III randomized multicentric trial

**DOI:** 10.1186/s13063-019-3676-x

**Published:** 2019-10-28

**Authors:** Francesco Cellini, Stefania Manfrida, Francesco Deodato, Savino Cilla, Ernesto Maranzano, Stefano Pergolizzi, Fabio Arcidiacono, Rossella Di Franco, Francesco Pastore, Matteo Muto, Valentina Borzillo, Costanza Maria Donati, Giambattista Siepe, Salvatore Parisi, Antonia Salatino, Antonino D’Agostino, Giampaolo Montesi, Anna Santacaterina, Vincenzo Fusco, Mario Santarelli, Maria Antonietta Gambacorta, Renzo Corvò, Alessio Giuseppe Morganti, Valeria Masiello, Paolo Muto, Vincenzo Valentini

**Affiliations:** 1 Fondazione Policlinico Universitario “A. Gemelli” IRCCS, UOC di Radioterapia Oncologica, Dipartimento di Diagnostica per Immagini, Radioterapia Oncologica ed Ematologia, Rome, Italy; 2 Radiotherapy Unit, Fondazione di Ricerca e Cura ‘Giovanni Paolo II’- Catholic University of Sacred Heart, Campobasso, Italy; 3 Medical Physic Unit, Fondazione di Ricerca e Cura ‘Giovanni Paolo II’, Catholic University of Sacred Heart, Campobasso, Italy; 4 Radiotherapy Oncology Centre, “S. Maria” Hospital, Terni, Italy; 5 Dipartimento di Scienze biomediche, odontoiatriche e delle immagini morfologiche e funzionali, Università di Messina, Messina, Italy; 6 Radiation Oncology Unit, Istituto Nazionale Tumori - IRCCS - Fondazione G. Pascale - Naples, Naples, Italy; 7 Fondazione Muto Onlus, Via Taverna Rossa 169/171 Casavatore, 80020 Napoli, Italy; 8 Department of Clinical Medicine and Surgery, Federico II University Medical School of Naples, Naples, Italy; 9 Radiation Oncology Unit, Department of Experimental, Diagnostic and Specialty Medicine, University of Bologna, S. Orsola-Malpighi Hospital, Bologna, Italy; 10 Unit of Radiation Therapy of IRCCS “Casa Sollievo della Sofferenza”, San Giovanni Rotondo, Italy; 11 Unit of Radiation Oncology, Fondazione del Piemonte per l’Oncologia/Institute for Cancer Research and Treatment of Candiolo (IRCCS), Torino, Italy; 12 Radiotherapy Unit, Humanitas, Catania, Italy; 13 Radiotherapy Unit ULSS5, Rovigo, Italy; 14 Operative Unit of Radiotherapy, Azienda Ospedali Riuniti Papardo-Piemonte Messina, Contrada Papardo, Messina, Italy; 15 Radiotherapy Oncology Department, IRCCS CROB, Rionero In Vulture, Italy; 16 Radiotherapy Unit, Ospedale San Camillo de Lellis, Rieti, Italy; 17 Università Cattolica del Sacro Cuore, Istituto di Radiologia, Roma, Italy; 18 Radiation Oncology Department, University of Genoa (DISSAL) and IRCCS Ospedale Policlinico San Martino, Genoa, Italy

**Keywords:** Bone metastases, Pain control, Simultaneous integrated boost, Randomised controlled trial

## Abstract

**Background:**

Palliative antalgic treatments represent an issue for clinical management and a challenge for scientific research. Radiotherapy (RT) plays a central role. Techniques such as stereotactic body radiotherapy (SBRT) were largely investigated in several phase 2 studies with good symptom response, becoming widely adopted. However, evidence from randomized, direct comparison of RT and SBRT is still lacking.

**Methods/design:**

The PREST trial was designed as an interventional study without medicinal treatment. It is a phase 3, open-label, multicentric trial randomized 1:1. Inclusion criteria include painful spinal bone metastases presenting with a pain level > 4 (or > 1 if being treated with an analgesic) on the Numeric Rating Scale (NRS); expected intermediate/high prognosis (greater than 6 months) according to the Mizumoto prognostic score; low spine instability neoplastic score (SINS) sores (< 7); magnetic resonance imaging (MRI) assessment of the bulky lesion. Patients will be assigned to either standard conventional radiotherapy involving 4 Gy × 5 fractions (fx) to the whole involved vertebra or SBRT by intensity modulated radiotherapy with simultaneous integrated boost (IMRT-SIB) involving 7 Gy × 3 fx to the whole involved vertebra + 10 Gy × 3 fx on the macroscopic lesion (gross tumor volume (GTV)). In the experimental arm, the GTV will be contoured by registration with baseline MRI.

**Discussion:**

The primary endpoint is overall pain reduction, defined in terms of variation between baseline and 3-month evaluation; pain will be measured using the NRS. Secondary endpoints include pain control duration; retreatment rates (after a minimum interval of 1 month); local control assessed with RECIST criteria; symptom progression free survival; progression-free survival; overall survival; and quality of life (at 0, 30, and 90 days). Accrual of 330 lesions is planned. The experimental arm is expected to have an improvement in overall pain response rates of 15% with respect to the standard arm (60% according to Chow et al. (Int J Radiat Oncol Biol Phys. 82(5):1730–7, 2012)).

**Trial registration:**

ClinicalTrials.gov, NCT03597984. Registered on July 2018.

**Electronic supplementary material:**

The online version of this article (10.1186/s13063-019-3676-x) contains supplementary material, which is available to authorized users.

## Background

The PREST trial (NCT03597984) includes patients with spinal bone metastases from solid tumors which present with an intermediate or high level prognosis (i.e., > 6 months) according to the Mizumoto score (i.e., classes A + B) [1]. These particular cases are often debated with regard to whether they should receive conventional antalgic radiotherapy or higher radiotherapy (RT) doses in order to better control symptoms. Technological improvements (e.g., stereotactic body radiotherapy (SBRT)) [2, 3] and advanced treatment planning (e.g., intensity modulated RT (IMRT)) [4] can enhance RT treatment efficacy by dose escalation to offer more effective pain (and potentially disease) control without compromising the organ at risk (OAR) through toxicity.

The aim of this randomized, multicentre, prospective trial is to evaluate the efficacy, in terms of pain control, of an unconventional RT fractionation (delivered by SBRT) against the standard one. The trial will enroll patients with intermediate-to-high prognosis (i.e., superior to 6 months) according to the Mizumoto prognostic score [1] and structural stability (according to the Spine Instability Neoplastic Score (SINS)) over a threshold of 7 [5]. Highlights of this study include the high level of treatment customization through both accurate selection and ultra-conformed RT planning and the reduced number of sessions (i.e., three instead of the gold standard) which patients will undergo, potentially limiting discomfort.

## Methods/design

### Aims

The PREST trial aims to assess whether RT for bone metastases administered by IMRT through a simultaneous integrated boost (SIB) can control pain symptoms better than fractionated standard three-dimensional conformal radiotherapy (3D-CRT). Providing better pain control is the crucial test for any new technique introduced in the palliative antalgic setting. Multicentre recruitment will allow more precise assessment of the intervention, with the aim of demonstrating that implementing RT in this setting is both feasible and safe among the centers. Secondary aims are to assess potential benefits for disease control, including oncological outcomes. Moreover, prolonged response to antalgic RT can avoid retreatment, thus improving quality of life in these individuals, which is an important goal.

### Overview of the design

**Fig. 1:**
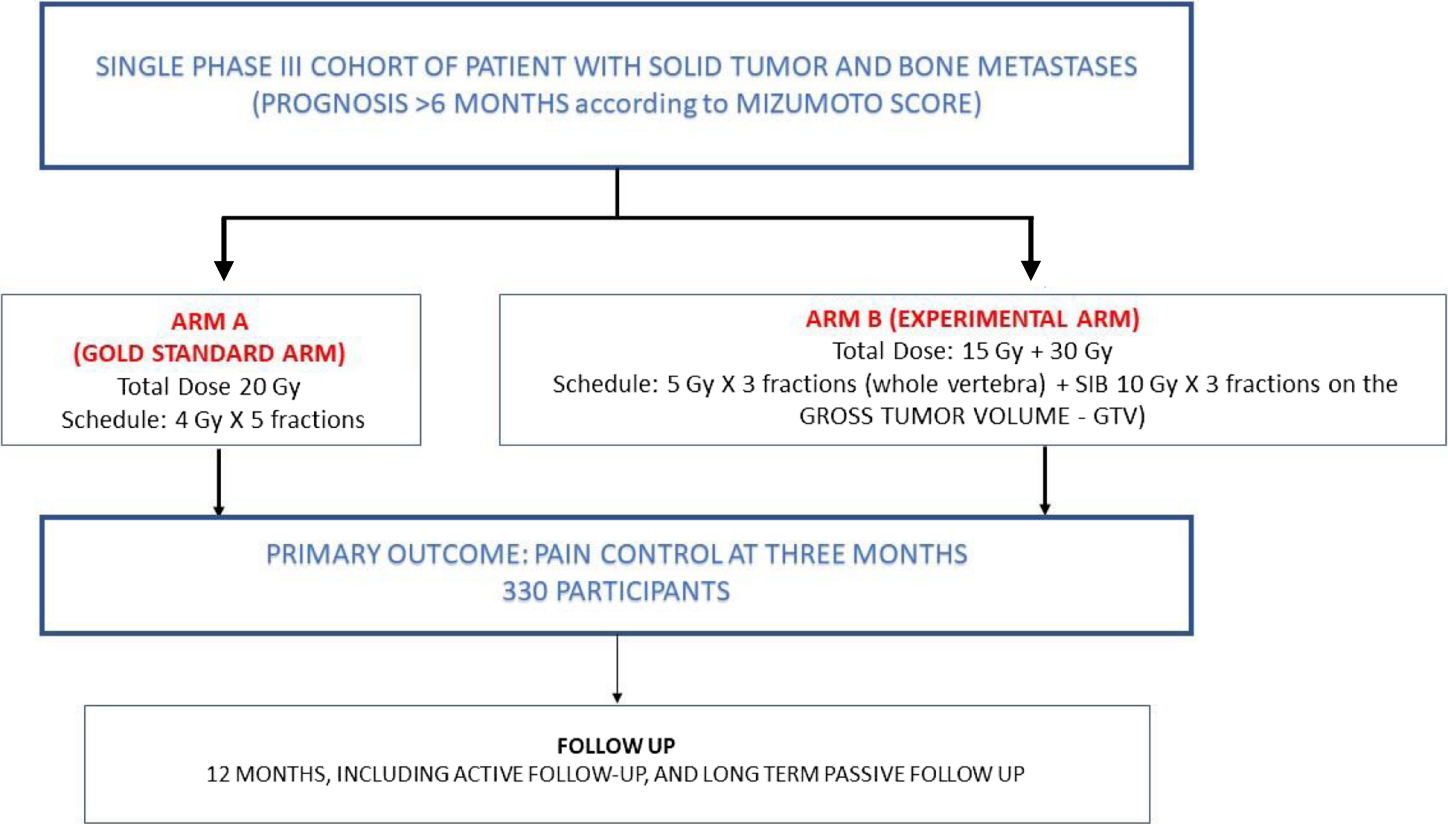
PREST trial (NCT03597984) schema

The PREST trial investigates the use of an advanced technique to administer 7 Gy × 3 fx (to the whole vertebra) plus SIB of 10 Gy × 3 fx to the macroscopic lesion (gross tumor volume (GTV)) defined by magnetic resonance imaging (MRI), compared to 4 Gy × 5 fx on the whole vertebra (standard arm) in patients with bone metastases from solid tumor and intermediate or high level prognosis > 6 months according to the Mizumoto score. Further details of the rationale for this design are provided in the “Discussion” section. Figure 1 shows a summary schema for the trial.

### Participants

Participants entering the PREST trial are affected by a solid tumor with histologically confirmed diagnosis and associated bone metastases. The estimated prognosis is intermediate or high (i.e., > 6 months) according to the Mizumoto prognostic score. Eligibility criteria are summarized in Table 1.

**Table 1:** Summary of eligibility criteria

PREST trial eligibility criteria	
Inclusion criteria	Exclusion criteria
• Patients diagnosed with spinal bone metastases from solid, uncomplicated tumor • Primary or secondary tumor histology related to the treatment lesion • Patients aged > 18 years • Obtained informed consent • ECOG 0–2 • Symptomatic patients at the treatment site (NRS ≥ 4) • Symptomatic patients at the treatment site (NRS 1–3) assuming opioid therapy ongoing for more than 3 days • Spine Instability Neoplastic Score (SINS) < 7 • Prognosis > 6 months according to Mizumoto Prognostic Score (i.e., classes A and B) • Spinal metastases verified at MRI, including the sites to be enrolled • No more than three non-contiguous spinal segments (e.g., separated by at least two metamers) involved in the study	• Unable to assign specific NRS for each CTV to be enrolled • Unable to express autonomous consent to therapies • Pregnancy • Patient in hospice or with prognosis < 6 months • Unavailability forecast for follow-up • Absence of MRI pre-treatment study • Unable to maintain the treatment position for SBRT • Previous radiotherapy at the same site or at the level of adjoining metameres (higher or lower than the one to be enrolled) • Previous radiometabolic therapy • Previous enrolment of the same patient for three irradiated lesions • Epidural compression of the spinal cord or of the cauda equina • Injuries affecting > 25% of the medullary canal and/or a distance < 5 mm from the medulla or from the cauda • Injuries with indication of surgical stabilization • Chemotherapy or target therapy within the previous 7 days and 7 days after SBRT

### Registration

The PREST trial is promoted by Fondazione Policlinico Gemelli—IRCCS in Rome (Italy). The local Ethics Committee has approved the protocol. Each interested center will submit the protocol to its ethics committee for approval before accrual. After approval, the center will receive a dedicated electronic case report form (CRF). Eligible participants who have provided consent and meet the inclusion criteria are anonymously registered on the CRF by assigning a numerical code.

### Randomization

Following the assessment for eligibility and after informed consent is signed, eligible participants are randomized by phone to the promoting centre. Participants undergo a blind randomization using minimization algorithms based on key prognostic factors, incorporating a random element. Patients are allocated in a 1:1 ratio to either gold standard radiotherapy treatment or interventional SIB radiotherapy treatment.

### Radiotherapy

CT simulation will be carried out with custom immobilization support for each patient (Aquaplast® head mask and/or vacuum mattresses). For target delineation, co-registration with MRI will be performed using the Velocity® application. The GTV is defined as the visible lesion on MRI imaging. In the experimental arm, two volumes will be defined: PTV1, including GTV plus a 2-mm isotropic margin; and PTV2, including total vertebra plus a 2-mm isotropic margin. In the standard arm, a single volume will be contoured: PTV1 (total vertebra plus a 1-cm isotropic margin).

In the experimental arm, both the spinal canal and spinal cord will be defined on MRI imaging, and their cranial and caudal margin will be the cranial margin of superior vertebra and caudal margin of inferior vertebra, respectively. The dose constraint limit for the spinal canal will be D_max_ < 15 Gy, while for the spinal cord it will be considered both D_max_ < 10 Gy [6] and D_0.035_. For cauda, dose constraints considered will be Dmax < 24 Gy and threshold dose of 21.9 Gy to less < 5 cc [6]. Moreover, in the SIB cohort, PTV coverage should be 95% of the prescribed dose at 95% of the defined volume. Major deviation for PTV2 will be < 77% of the dose prescribed at 95% of the volume, while minor deviation will be < 84% of the prescribed dose at 95% of the volume. For PTV1, major deviation will be defined as < 79% of the prescribed dose at 95% of the volume, while minor deviation will be < 84% of the prescribed dose at 95% of the volume.

Reporting of dose prescription will be done according to ICRU 83 for the experimental arm and according to ICRU 62 for the standard arm. Furthermore, as security criteria, all treatment plan validation will rely on QUANTEC and AAPM reports for organ at risk (OAR) constraints [6, 7].

### Follow-up

Patients are assessed according to International Bone Metastases Consensus Working Party [8] criteria. Pain control will be assessed at every visit according to IBMC criteria (Additional file 1). IBMC criteria are based on pain level and ongoing antalgic opioid therapies. Pain level will be defined using the 11-point NRS. NRS score subscales evaluated will be:

- 0: no pain (better outcome)
- 1–3: mild pain
- 4–6: moderate pain
- 7–10 severe pain (worse outcome)

Opioid therapies will be quantified according to Oral Morphine Equivalent Dose (OMED). The visit schedule is 15 days, 30 days, 3 months, 6 months, and 12 months after the end of radiotherapy. At every visit, patients will undergo quality of life measurement using the QLQ-C15-PAL and QLQ– BM22 questionnaires [9]. Other outcomes that will be tested during follow-up visits are rate of retreatment for non-responding patients, pain control duration, local control, symptom progression-free survival, progression free survival, and overall survival. Long-term passive follow-up data will be obtained from routinely collected healthcare databases for at least 10 more years.

For participants that are registered but do not go on to be randomized, active participation in the trial will end at that time. However, passive follow-up will continue via routinely collected healthcare datasets where consent for this has been obtained. The trial assessment schedule for each arm is aligned with standard practice where possible to ensure they can be implemented easily. This is balanced with the need to ensure appropriate monitoring of patients on trial treatment and assessment of outcome measures. The trial follow-up schedules are available in Additional file 2.

### Toxicity management

Participants that experience radiotherapy-related severe toxicity, defined as ≥ Grade 3 Common Terminology Criteria for Adverse Events (CTCAE v4) [10], will be signaled in the study final report. Grade 1 and 2 toxicity are considered normal in clinical practice, but they will also be reported.

## Discussion

### Outcomes

One-month pain control analysis will take place 90 days after the end of RT of the last patient. Primary and secondary outcome measures are available in Table 2.

**Table 2:** Summary of primary and secondary endpoints

Endpoint definitions and measurement	
Primary outcome measure	
Pain control (efficacy and pain; time frame 3 months) Overall pain control measured according to IBMC (complete response + partial response events)	
Secondary outcomes measures	
• Pain control duration (efficacy and pain; time frame 12 months after end of radiotherapy) Interval from the end of the RT to relapse of the symptom • Rate of retreatments (efficacy; time frame 12 months after end of radiotherapy) Interval from the end of the RT to the start of retreatment • Local control (efficacy; time frame 3, 6, and 12 months from the end of radiotherapy) Control of local disease with diagnostic exams according to RECIST 1.1 criteria • Symptom progression-free survival (SPFS) (efficacy and pain); time frame 12 months after end of radiotherapy) Interval from the end of radiotherapy and progressive disease with symptoms according to the criteria of Chow et al. in 2012 [8] • Progression-free survival (efficacy; time frame 12 months) Interval from the end of radiotherapy and new disease progression • Overall survival (efficacy; time frame 12 months) Interval between the end of radiotherapy and death • Quality of life (efficacy and quality of life; time frame first visit, 1 month, and 3 months after the end of radiotherapy) Quality of life score according to European Organization for Research and Treatment of Cancer (EORTC) QLQ-C15-PAL and QLQ– BM22 questionnaires	

Local control and overall survival will also be assessed as secondary outcomes in all participants for 10 years. The longer follow-up and large sample size associated with this analysis will enable any long-term benefits of RT with IMRT-SIB to be determined, including number of retreatments. Consideration of rates of serious toxicity (and particularly serious myelotoxicities), as well as other secondary health outcomes, alongside the efficacy results will be particularly important in these analyses in order to provide a holistic assessment of the potential risks and benefits associated with a different schedule of RT administration.

### Statistical considerations and sample size

Primary analysis will compare the response in terms of reduction of pain symptomatology from bone metastases, comparing the conformational RT (3D-CRT) administered in conventional fractionation versus IMRT-SIB. The study involves the enrolment of 330 patients divided into two groups of 165 patients in each of the two study arms. Arm A (standard) will recieve 4 Gy × 5 fx; arm B (experimental) will receive SIB 7 Gy × 3 fx (whole vertebra) + 10 Gy × 3 fx on the GTV (defined by MRI). The expected difference between the two treatments for one-month pain control in terms of overall response rates is 15% more in the experimental arm compared to the standard arm (60%). The calculation of the sample size considered a 95% CI with a coefficient α of 0.05 and a drop-out rate of 10%.

### Ethics considerations

The trial will be conducted in compliance with the approved protocol, the Declaration of Helsinki 2008, the principles of Good Clinical Practice (GCP), and Italian National Normative for clinical experimentation. Upon signing the protocol, every investigator gives consent for the procedure and instructions in the protocol and run the study according to GCP, Declaration of Helsinki, and National Normative. Every amendment of the study will be registered and submitted to the Ethics Commission. The PREST trial protocol and its attached material have been approved by the Ethics Commission of Fondazione Policlinico Gemelli IRCCS of Rome (Italy). Every participant center needs to submit the PREST trial protocol to their respective ethics commission before enrolling patients.

### Quality assurance

Dose escalating in radiotherapy requires a quality assurance procedure. In our study protocol we guarantee this procedure by:

- Diagnostic imaging: MRI imaging before treatment for GTV contouring; follow-up MRI imaging at 3 months is recommended but not mandatory
- Personalized set-up system: the use of personalized set-up systems is considered mandatory
- Planning verification: every single treatment plan needs to respect coverage and constraints indicated in the protocol; before enrolling, every single center should participate in dummy studies to confirm its treatment planning possibilities
- Image-guided radiotherapy (IGRT): Cone-beam daily acquisition before treatment is mandatory to assure good IGRT. A six-degree-of-freedom couch is recommended but not mandatory for set-up error correction

### Final considerations

Palliative antalgic oncological treatments represent a serious problem for both clinical management and scientific research. However, they involve an ever-increasing number of patients due to the increased incidence of cancer in all its phases and the potential chronicity of illness linked to new therapies. The use of palliative RT treatments potentially involves up to 40% of patients in a radiation oncology center. RT is commonly used in palliative treatment for symptomatic bone metastases [11], being an effective treatment for improving symptoms and, consequently, the quality of life of these patients.

Ideally, this treatment should be as short as possible, re-directing patients either to systemic therapies or to home care or long-term care systems (e.g., hospices). In order to deliver a clinically effective dose in a short period, hypofractionated regimens must be used. SBRT is a technique of RT allowing delivery of a high equivalent biological dose in a highly conformed manner, with a favorable toxicity profile [3], generally in a few fractions (fx). The possibility of using special techniques such as SBRT in the palliative antalgic setting for bone metastases has been investigated in several phase 2 studies, with good results in terms of symptom response [12–17]. In order to better manage the toxicity profile of such hypofractioned regimens (mainly related to risk of vertebral fracture [18]) further studies [19, 20] have suggested the possibility of using a hypofractionated regimen over the entire bone compartment and going to over-dose with a stereotactic regimen only for the macroscopically visible disease from instrumental examinations. In patients with more favorable prognosis, this regimen could improve the possible onset of acute and late complications, while increasing the RT dose to the bulky lesion.

Literature indications about preferred RT schedules are available, although there is no globally coded and unique clinically applied therapeutic standard for prescription [8, 21]. The most commonly applied conventional radiation treatment schedules include i) 8 Gy in 1 fx, ii) 20 Gy in 5 fx, iii) 30 Gy in 10 fx [22].

Although achieving similar rates of overall pain control, multiple fractionation schedules have been reported to provide better symptom control over time, lower the rate of need for retreatment, and improve bone stability profiles over the single fx approach, in case of a patient’s longer life expectation. Single fraction (8Gy) RT should be preferred for patients with inferior prognosis. Fractionated schedules are therefore often preferred for patients with better prognosis (i.e., > 6 months): the most widely adopted and supported by expert consensus is represented by 20Gy delivered in 5 fx of 4 Gy [23]. Routine use of prognostic scores to characterize life expectancy and define the most appropriate treatment regimen is very rarely applied in everyday clinical practice. Some randomized trials [8, 12] are underway investigating the role of SBRT compared to conventional approaches for these patients, though not all of them through patient selection by validated prognostic scores. At the present time, a study by Guckenberger et al. (NCT02800551, DOSIS RCT, https://clinicaltrials.gov/ct2/show/NCT02800551) is directly comparing a schedule administering 20 Gy in 5 fx to SBRT-SIB given with 5 or 10 fx.

The Ethics Commission approval for the PREST trial was given on May 2018. Registration on ClinicalTrial.gov was approved on July 2018. The PREST trial was provided grants for insurance costs by Fondazione Policlinico Gemelli IRCCS. The first patient is expected to be recruited on January 2020.

## Conclusions

The PREST trial will provide insight on efficacy of a hypofractionated SBRT IMRT-SIB in pain control with respect to gold standard fractionation. Highlights of this study include personalization by prognostic score stratification, selection based on imaging-driven stability scores, ultra-conformed RT planning, and lower number of RT sessions. To our knowledge, this is the first study design of its kind. The results will clarify if this highly personalized approach would be practice-changing in the setting of metastatic bone patients.

### Trial status

Patient recruitment not completed.

## Additional files

Additional file 1: Description of data: IBMC criteria for assessing pain control. (DOCX 18 kb)

Additional file 2: Description of data: Follow-up control schedule. (DOCX 20 kb)

## Data Availability

Not applicable.

## References

[1] Mizumoto M (2008). Prognostic factors and a scoring system for survival after radiotherapy for metastases to the spinal column: A review of 544 patients at Shizuoka Cancer Center Hospital. Cancer.

[2] Blomgren H, Lax I, Naslund I, Svanstrom ER (1995). Stereotactic high dose fraction radiation therapy of extracranial tumors using an accelerator. Clinical experience of the first thirty-one patients. Acta Oncologica.

[3] Correa R, Salama J, Milano M, Palma ED (2016). Stereotactic body radiotherapy for oligometastasis: Opportunities for biology to guide clinical management. Cancer J.

[4] Xu C (1995). Intensity-modulated arc therapy with dynamic multileaf collimation: an alternative to tomotherapy. Phys Med Biol.

[5] Fisher C (2010). A novel classification system for spinal instability in neoplastic disease: an evidence-based approach and expert consensus from the Spine Oncology Study Group. Spine (Phila Pa 1976).

[6] Benedict SH (2010). Stereotactic body radiation therapy: The report of AAPM Task Group 101: Stereotactic body radiation therapy: The report of TG101. Med Phys.

[7] Bentzen SM (2010). Quantitative Analyses of Normal Tissue Effects in the Clinic (QUANTEC): An introduction to the scientific issues. Int J Radiat Oncol Biol Phys.

[8] Chow E (2012). Update of the International Consensus on Palliative Radiotherapy Endpoints for Future Clinical Trials in Bone Metastases. Int J Radiat Oncol Biol Physi.

[9] Lam K (2013). Predictive factors of overall well-being using the EORTC QLQ-C15-PAL extracted from the EORTC QLQ-C30. J Palliat Med.

[10] National Cancer Institute (NCI), Common Terminology Criteria for Adverse Events (CTCAE) Version 5.0, November 27, 2017. https://ctep.cancer.gov/.

[11] Furfari A (2017). Genetic biomarkers associated with pain flare and dexamethasone response following palliative radiotherapy in patients with painful bone metastases. Ann Palliat Med.

[12] van der Velden JM (2016). Comparing conVEntional RadioTherapy with stereotactIC body radiotherapy in patients with spinAL metastases: study protocol for an randomized controlled trial following the cohort multiple randomized controlled trial design. BMC Cancer.

[13] Murai T (2014). Intensity modulated stereotactic body radiation therapy for single or multiple vertebral metastases with spinal cord compression. Pract Radiat Oncol.

[14] Braam P, Lambin P, Bussink EJ (2016). Stereotactic versus conventional radiotherapy for pain reduction and quality of life in spinal metastases: study protocol for a randomized controlled trial. Trials.

[15] Deodato F (2014). Stereotactic radiosurgery (SRS) with volumetric modulated arc therapy (VMAT): interim results of a multi-arm phase I trial (DESTROY-2). Clin Oncol (R Coll Radiol).

[16] Ryu S (2011). RTOG 0631 Phase II/III study of image-guided stereotactic radiosurgery for localized spine metastases: Phase II results. Int J Radiat Oncol Biol Phys.

[17] Sprave T (2018). Randomized phase II trial evaluating pain response in patients with spinal metastases following stereotactic body radiotherapy versus three-dimensional conformal radiotherapy. Radiother Oncol.

[18] Faruqi S (2018). Vertebral compression fracture after spine stereotactic body radiation therapy: A review of the pathophysiology and risk factors. Neurosurgery.

[19] Lee YK, Bedford JL, McNair HA, Hawkins EMA (2013). Comparison of deliverable IMRT and VMAT for spine metastases using a simultaneous integrated boost. Br J Radiol.

[20] Wu Q, Yoo S, Kirkpatrick J, Thongphiew D, Yin EF (2009). Volumetric arc intensity-modulated therapy for spine body radiotherapy: comparison with static intensity-modulated treatment. Int J Radiat Oncol Biol Phys.

[21] Chow E, Wu J, Hoskin P, Coia L, Bentzen S, Blitzer EP (2002). International consensus on palliative radiotherapy endpoints for future clinical trials in bone metastases. Radiotherapy and Oncology.

[22] Rich S (2018). Update of the systematic review of palliative radiation therapy fractionation for bone metastases. Radiother Oncol.

[23] Chow R (2017). Efficacy of multiple fraction conventional radiation therapy for painful uncomplicated bone metastases: A systematic review. Radiother Oncol.

