# Supplementary material for: Pain REduction with bone metastases STereotactic radiotherapy (PREST): A phase III randomized multicentric trial
Source: Trials. 2019 Oct 28;20:609. doi: 10.1186/s13063-019-3676-x (PMC6816218; doi:10.1186/s13063-019-3676-x)
Supplement: Supplementary file 1 — Description of data: IBMC criteria for assessing pain control. (DOCX 18 kb) [file 13063_2019_3676_MOESM1_ESM.docx]

**Additional file 1 IBMC table Criteria for assessing pain control**

| DISTRIBUTION ACCORDING TO RESPONSE CATEGORIES ACCORDING TO THE INTERNATIONAL CONSENSUS WORKING PARTY GUIDELINES [22] | |
| --- | --- |
| COMPLETE RESPONSE (CR)  “A pain score of 0 at treated site with no concomitant increase in analgesic intake (stable or reducing analgesics in OMED)” | **Pain Control achieved** |
| PARTIAL RESPONSE (PR)  “Pain reduction of 2 or more at the treated site on a scale of 0 to 10 scale without analgesic increase, or analgesic reduction of 25% or more from baseline without an increase in pain” | **Pain Control achieved** |
| PAIN PROGRESSION (PP)  “Increase in pain score of 2 or more above the baseline at the treated site with stable OMED, or an increase in OMED compared with baseline with the pain score stable or 1 point above baseline” | **Pain Control NOT achieved** |
| INDETERMINATE RESPONSE  “Any response that is not captured by the complete response, partial response or pain progression definition” | **Pain Control achieved** |
