# Supplementary material for: Pain REduction with bone metastases STereotactic radiotherapy (PREST): A phase III randomized multicentric trial
Source: Trials. 2019 Oct 28;20:609. doi: 10.1186/s13063-019-3676-x (PMC6816218; doi:10.1186/s13063-019-3676-x)
Supplement: Supplementary file 2 — Description of data: Follow-up control schedule. (DOCX 20 kb) [file 13063_2019_3676_MOESM2_ESM.docx]

**Additional file 2 Follow up control schedule**

| - Schedule | V0  (First evaluation) | RT | V1  (evaluation at 15 days from end of RT) | V2  (evaluation at 30 days from end of RT) | V3  (evaluation at 90 days from end of RT) | V4  (evaluation at 6 months from end of RT) | V5  (evaluation at 12 months from end of RT) | V6  (evaluation at 18 months from end of RT) |
| --- | --- | --- | --- | --- | --- | --- | --- | --- |
| Eligibility Criteria evaluation | X |  |  |  |  |  |  |  |
| Sign of Informed Consent | X |  |  |  |  |  |  |  |
| Oncological anamnesis (with OMED on going registration) | X |  | X^*^ | X^*^ | **X** | X^*^ | X^*^ | X^*^ |
| Clinical visit (with NRS registration for every site to be treated) | X |  |  |  | **X** |  |  |  |
| Evaluation of previous and ongoing therapies | X |  |  |  |  |  |  |  |
| MRI imaging | X |  |  |  |  |  |  |  |
| Radiotherapy |  | X |  |  |  |  |  |  |
| Phone and/or outpatient visits (with NRS and OMED follow up) |  |  | X | X | X | X | X | X |
| Imagin follow-up (MRI, CT, bone scintigraphy) |  |  |  |  | X^*^ |  |  |  |
| Side Effect Evaluation |  |  | X^*^ | X^*^ | **X** | X^*^ | X^*^ | X^*^ |
| Quality of Life Evalulation | X |  | X | X | **X** | X | X | X |
| End of active monitoring |  |  |  |  |  |  |  | X |
| ^*^optional | | | | | | | | |
